# Allegheny County Medical Society

**Published:** 1890-05

**Authors:** 


### ALLEGHENY COUNTY MEDICAL SOCIETY.

Special Meeting, March 13, 1890.

W. E. Johnston, M. D., Vice-President, in the chair.

Dr. A. S. Daggette reported a case of a woman, seven months pregnant, who aborted four days after a fall. The child lived about forty minutes ; the skin on its lower extremities and on the lower part of the abdomen had the appearance of being parboiled, and peeled off on handling, leaving large, livid, mottled spots. This macerated condition existed only in the localities named. The after-birth was normal. No history of syphilis.

Dr. Macfarlane thought the condition undoubtedly due to syphilis.

Dr. F. H. Edsall reported a case of

### MALFORMATION OF THE EXTERNAL AUDITORY APPARATUS.

The patient, Ida G., aged 8, is of Jewish parentage, is delicate and rather anæmic. The immediate cause of consultation was constant pain in the right ear. This pain was neuralgic in character, although there was shown, upon examination, to be a recent slight purulent otitis existing in this ear. The otalgia had been of much longer standing, showing that no connection existed between the two. This ear presented no appearances different from those customarily seen in cases of acute otitis media; it was normal in all its parts in as far as its development is concerned.

The left ear was a marked instance of arrest of development during fœtal life. The entire auricle is small, probably not more than half the size of its fellow, and its several parts show a varying degree of imperfection in development. The helix, particularly near the upper margin of the auricle, is almost wanting, and the fossa of the helix is correspondingly imperfectly developed. The anti-helix is also scarcely to be traced and the fossa of the anti-helix is consequently lacking also, and there is no trace of anything resembling a tragus. The anti-tragus and lobule are more perfectly or, rather, less imperfectly developed than the other parts, but these, too, fall short of full development. The concha is very small and shallow. An external auditory maetus is lacking and the only indication of the site of such an opening is a shallow depression, probably about one millimetre in depth, in the skin over what would otherwise be the outer opening of the external auditory canal. Upon making firm pressure over the center of this depression it is possible to deepen it somewhat, indicating thus that there exists beneath at least a cartilaginous canal. Hearing on this side of the head, as was to be expected, is entirely absent. Neither the ticking of the watch nor of Politzer's "Hoermesser" can be heard even on close contact, according to patient's statement. Absolute reliance could not be placed on patient's statements in regard to this, as she is timid and is, moreover, somewhat dull of comprehension. But as it usually is in such cases, that the middle ear and Eustachian tube share in the defect in development, it is more than probable that her statements were correct. The hard palate is, not infrequently, affected by the same arrest of development which overtakes the auditory apparatus, but in this instance nothing of the kind was observed.

As to what is best to do in a case like this, I think there is but one thing to be said; let it alone. Surgical interference would be meddlesome, for, aside from the difficulty which would be experienced in preventing an artificial opening from closing up again, there would be little probability of such artificial canal proving of any benefit. The reason for this can be readily understood when we consider that, according to Virchow, these cases are due to a disturbance in the process of closure of the pharyngeal cleft, early in fœtal life. Therefore, inasmuch as the external ear, the tympanic cavity and the Eustachian tube are all developed either from the first pharyngeal fissure or the first and second pharyngeal arch, it usually follows that marked imperfections in the development of the external auditory apparatus are accompanied by corresponding defects in the other portions of the auditory apparatus developed from the fœtal structures named. In consequence of this, even if an open pathway for sound-waves were successfully maintained, the results, in cases like the one I have named, would probably be nil, due to defects in the tympanum.

Moreover, in a patient as old as this one, I am inclined to believe that even were the tympanum fully developed, hearing would at best be very imperfect, because of the long-continued disuse of the organ, just as we find a squinting eye becoming amblyopic in the course of time from lack of use, although I have no data bearing on the subject of the effect of disuse on the auditory nerve.

Cases like the one I have related, while not extremely rare, are sufficiently so to make them of interest as curiosities, although they are of little practical value.

DR. LIPPINCOTT.—Four patients presented themselves at my office within three weeks with foreign bodies which had entered the eye, and in the first three cases I made an effort at extraction with the magnet. In two I succeeded in withdrawing the steel from the neighborhood of the retina and the optic nerve with the magnet, and there was a recovery of a moderate degree of vision. In the third case, suppuration took place subsequent to the removal of the steel; the steel was removed at the operation and everything appeared to go well for about six days, and then irritation appeared and suppuration subsequently occurred. In the fourth case the patient was so positive that nothing had entered his eye that, although all the signs pointed in that direction, I did not feel like taking the responsibility of urging an operation, because if steel was not in his eye any operation would have interfered with its recovery. The man went to the point of refusing an operation. He went home and returned in two weeks with severe inflammation of the eyeball. I enucleated the eye and found a large piece of steel.

The cases are not specially instructive, except in so far as to suggest that if the foreign body happens to be of steel or iron, or something that will respond to the magnet, an effort should perhaps generally be made to withdraw it. There are cases, of course, in which that would scarcely be allowed, as, from the nature of the injury, an inflammation of the eye might be excited, which would involve the other eye. But if the wound happens to be not in a very vital point, an effort should be made to withdraw the foreign body, and in that my experience lately would seem to show a certain degree of success can be anticipated. The last case was instructive, as showing how little dependence is to be placed upon the opinion of a patient. The patient generally thinks that nothing went into the eye; in nine cases out of ten I do not believe the patient thinks a foreign body has entered the eye. The man in my case was certain.

DR. ALLYN.—I have had a case quite similar about two months ago, which has bothered me a good deal, owing to the lack of activity in the inflammation. The man was struck by a fragment of steel, which punctured the cornea, and cut a hole directly back through the iris. He reached my office within two hours after the injury, when he was absolutely blind to all light; could not see a finger or anything. He maintained, as the doctor has said they all do, there was nothing in the eye; he had simply been struck in the eye, and a physician had removed the foreign substance. I told him it was my opinion the steel was in the eye, and asked him to inquire of the physician if it was really a metallic substance he had picked out, and on his return he told me it was nothing but a piece of dirt. I told him to come back in a day or two, giving a chance for inflammation. The next day he returned feeling perfectly comfortable, and has never had to this time a particle of pain. There was a small particle up in the anterior chamber which resembled metal covered with pus, and for two or three days I was uncertain whether or not it might be metal. Finally, under the treatment the particle completely cleared, leaving adhesion of the inner border of the iris to the anterior surface of the lens. He continued to get better, and maintained there was nothing in the eye; I strongly maintained there was something in it, but the patient wishing no operation, I left the case from time to time, the eye improving, but at the last examination I found a band reaching through the lens and striking backward into the center of the eye. At the end of that band I am sure there was a piece of metal.

DR. W. C. SHAW.—I was called recently to examine a man for life insurance, a very healthy man who said he had not had occasion for a family physician for himself, never having been sick. When he was about to go, I asked for a specimen of his urine. In the bottle the fluid looked as clear as crystal. I put the specimen in my pocket and came to the office and examined it and found it to have a specific gravity of 1012, and to be full of sugar. This is the only specimen of so low gravity and containing sugar I have ever had.

DR. LIPPINCOTT.—That is a very low specific gravity. I saw a case of saccharine diabetes in Philadelphia before I came to Pittsburg, and the man had lived twenty-five years with that sort of urine. Dr. Austin Flint, Sr., had told the man, twenty-five years before I saw him, that he would be dead within a year, but the man told me he was enjoying fairly good health at that time.

DR. LANGE.—Dr. Shaw's case is peculiar, inasmuch as urine with a specific gravity of 1012, full of sugar, is a rare specimen. In the text books and pocket manuals for the examination of urine, you will frequently find it stated that no urine of a specific gravity of less that 1012 can contain sugar. Dr. Shaw's case—the urine presenting a specific gravity of 1012 and full of sugar—is an exemplification that this statement is erroneous. I, myself, have more than once obtained specimens of less than 1020 specific gravity, which though not "full" contained some sugar.

DR. MCCANN.—The following case recently came under my observation, a German suffering from cancer which involved the rectum about three inches above the verge of the anus, in which the whole anterior wall was involved and fully three-fourths of the rectum, the disease extending fully two inches up the rectum. The patient suffering from the distress which attends this condition came under my care. The question as to excision of the rectum or colotomy came up. I determined to resort to another operation, one which does not involve the technique of a formidable excision but which is equally effective. The patient being etherized and placed in a proper position, I introduced my finger into the rectum as far as I could, and then with a Volkmann's spoon scraped out the growth slowly piece by piece, avoiding the wall of the bladder in front, the base of the bladder and guarding against entering the peritoneum. Thus by a careful and slow, not an elegant operation by any means, but a careful scooping out, just as is the habit in scooping out cancerous growth from the cervix uteri, I was able to remove the entire growth. In doing this, I did not destroy the external sphincter; I did not damage the wall of the bladder. I avoided opening the peritoneal cavity but I certainly cleaned away every portion of diseased tissue down to the peritoneum. The operation required a considerable length of time. Fortunately the sphincter was so easily dilated that I had abundance of room, and after having removed a portion of the mass was enabled by the use of Sim's speculum to hold back the posterior wall of the rectum, the portion least diseased, so that I had a very fair view, and drawing down the rectum I was enabled to get beyond the diseased area and into that portion of the rectum where I found the mucous membrane was normal. After having done this, the surface was carefully washed with an antiseptic solution and the bowel plugged with iodoform gauze. The bowels moved on the third day. The rectum was again washed out, plugged for a time with iodoform gauze, and afterwards allowed to remain without any treatment whatever. The result was that the distressing pain from which the patient suffered was relieved at once. Since that operation six months have elapsed. During all of this time, until quite recently, the patient has been free from pain. Recently, however, a new growth, or rather a recurrence of the growth, has appeared upon the anterior wall of the rectum. The patient has promised to come back for a repetition of the treatment. As a result of this operation, there was no incontinence of fæces. The external sphincter was left undisturbed. The internal sphincter was involved in the growth and removed; he had, however, control of the bowels. The operation did not result in contraction or stricture of the rectum. Recently I assisted a friend in the removal of a cancerous growth from the rectum in which a more formidable operation was undertaken.

The perineum was split from the point of the coccyx clear into the rectum, and then an effort was made to cut out the diseased tissue. A portion of the diseased tissue was removed, but the growth was so extensive that its complete removal with the knife had to be abandoned. It occurred to me at the time of that operation and inthinking over the matter since, that the safer operation is to simply scoop out all of the tissue possible, to clean away the diseased tissue, and to make your patient as comfortable as possible, because you cannot hope to remove a malady which is going to destroy him sooner or later.

